# Whole-exome sequencing for detecting inborn errors of immunity: overview and perspectives

**DOI:** 10.12688/f1000research.12365.1

**Published:** 2017-11-28

**Authors:** Barbara Bosch, Yuval Itan, Isabelle Meyts

**Affiliations:** 1 Department of Pediatrics, University Hospitals Leuven, Leuven, Belgium; 2 St. Giles Laboratory of the Human Genetics of Infectious Disease, Rockefeller University, New York, USA; 3 The Charles Bronfman Institute of Personalized Medicine, Icahn School of Medicine at Mount Sinai, New York, USA; 4 Department of Genetics and Genomic Sciences, Icahn School of Medicine at Mount Sinai, New York, USA; 5 Childhood Immunology, KULeuven, Leuven, Belgium

**Keywords:** primary immune deficiencies, inborn errors of immunity, whole-exome sequencing

## Abstract

The study of inborn errors of immunity is based on a comprehensive clinical description of the patient’s phenotype and the elucidation of the underlying molecular mechanisms and their genetic etiology. Deciphering the pathogenesis is key to genetic counseling and the development of targeted therapy. This review shows the power of whole-exome sequencing in detecting inborn errors of immunity along five central steps taken in whole-exome sequencing analysis. In parallel, we highlight the challenges for the clinical and scientific use of the method and how these hurdles are currently being addressed. We end by ruminating on major areas in the field open to future research.

## Introduction

Inborn errors of immunity (IEIs) or primary immune deficiencies (PIDs) are inherited defects leading to errors in one or more components of the immune system. The presentation of IEIs is variable, and phenotypes are as diverse as increased susceptibility to infection, auto-inflammation, autoimmunity, allergy, and malignancy. Ever since the description of the first IEI, Bruton agammaglobulinemia (1952), their study has been built upon (1) a thorough clinical description, (2) the elucidation of the crippled cellular pathway and molecular mechanisms, and (3) the genetic etiology. Bruton noticed complete absence of gammaglobulin in a child with recurrent pneumococcal sepsis and postulated a defect in the antibody response ^1^. Race and Sanger mapped the agammaglobulinemia locus to the X-chromosome ^2^, and ultimately the role of Bruton tyrosine kinase (BTK) in B-cell development was recognized ^3^. This three-step approach, translated in the contemporary detection of IEI by clinical phenotyping, testing a genetic hypothesis, and functionally validating a candidate variant, remains fundamental to date in both the clinical and research setting ^4^.

The tools available for identifying IEIs have evolved over time. The introduction of Sanger sequencing for detecting disease-causing mutations revolutionized medicine. It allows the generation of a high-quality sequence of up to 900 nucleotides and has a low error rate (0.001%) ^5^. With this technique, mutations in 223 genes, including *BTK*, have been shown to underlie IEI. However, Sanger sequencing is intrinsically low-throughput, time-consuming, and hypothesis-driven. Therefore, the prior identification of a region or a set of genes of interest (for instance, via linkage analysis, positional cloning, genetic homology analysis, or identification of the affected pathway) is essential for the identification of a novel disease-causing gene using Sanger sequencing. The introduction of next-generation sequencing (NGS) in 2010 allowed the genetic study of IEI by sequencing the whole-exome (WES) or the whole-genome (WGS) or the transcriptome (RNA-seq) or a combination of these. NGS techniques use massive parallel sequencing, allowing the generation of gigabases of genome-wide information in a single run and thus a fast and unbiased approach to identifying the etiology of a disease. This resulted in the identification of 47 novel IEIs in the last three years (2014–2016). Yet the length of reads is shorter and sequencing error rates are higher (up to 2%) ^5^. As these techniques became widely accessible and more affordable and started being used in scientific but also clinical settings, it became apparent that identifying the disease-causing variant in the pile of information generated by WES and WGS, respectively, requires a methodology much like the three-step approach elaborated above.

This review presents the recent advances in detecting the genetic origin of IEIs using NGS. We will discuss (1) the technical data acquisition, (2) the generation of a genetic hypothesis, (3) the variant-level interpretation, (4) the gene-level interpretation, and (5) the functional validation. We focus on WES, which allows the analysis of the exome: the DNA sequence encompassing all exons of protein coding genes, microRNA, small nucleolar RNA, and large intergenic noncoding RNA in the genome (around 2% of the patient’s genome) ^6^. WES is currently the most widely used NGS technique for the detection of IEIs. Extensive overviews of this approach have recently been published ^4, 7– 9^; therefore, we will elaborate only on the core components here along a structured flowchart. We will highlight the challenges for each of the five components described.

## Advances in testing a genetic hypothesis using whole-exome sequencing

### Whole-exome sequencing – generation and bioinformatic pipeline

The generation of a WES dataset starts with the polymerase chain reaction (PCR) amplification of the patient’s genomic DNA. The library of primers used for this PCR reaction is designed to span the entire human exome (capture step). The nucleotide sequences of the short PCR amplified pieces of DNA (amplicons) are made available for further analysis in a FASTQ output file. A FASTQ is a file that contains base call and quality information for all sequence reads that pass filtering. This is the common raw electronic data provided after sequencing ^10^. Subsequently, data are mapped to a genomic position (alignment) and stored in a Binary Alignment/Map (BAM) or CRAM file. BAM is a binary file that contains data about the reads’ alignment to the reference genome. BAM files are used for downstream annotations and reads visualizations ^11^. CRAM files are an alternative format to BAM, used in the 1000 Genomes Project ^12^.

**Figure 1.**
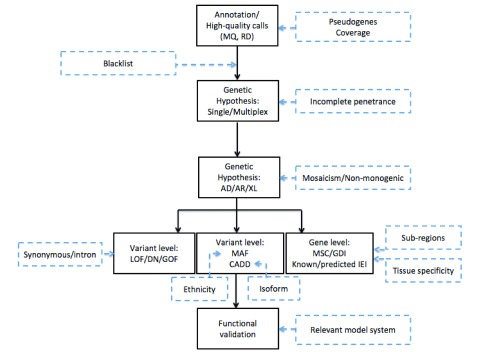
Approach to the use of whole-exome sequencing for the detection of inborn errors of immunity. A schematic overview of the different steps taken during whole-exome sequencing analysis (black boxes) with the challenges identified in recent research that need to be accounted for in future research (blue dashed boxes). AD, autosomal dominant; AR, autosomal recessive; CADD, Combined Annotation Dependent Depletion; DN, dominant negative; GDI, gene damaging index; GOF, gain of function; IEI, inborn errors of immunity; LOF, loss of function; MAF, minor allele frequency; MQ, Mapping Quality; MSC, mutation significance cutoff; RD, read depth; XL, X-linked.

Only high-quality reads are kept and compared with a human reference sequence. Nucleotides that differ between both sequences are “called” into a variant call format (VCF) file. The VCF also contains information on coverage or sequencing depth: the number of times a specific nucleotide is read. If around 50% or nearly 100% of the reads show a nucleotide differing from the reference genome, the position is called a heterozygous or homozygous variant, respectively. A sufficiently high coverage (typically, more than 20) is essential to discriminate a sequencing error from a true variant. Analysis of the sequencing depth can also be applied to detect somatic mosaicism where less than 50% of reads contain an alternative nucleotide; however, in the latter case, a greater read depth of at least 50 is recommended ^13^. WES typically generates over 100,000 high-quality variants per patient. At most, two of these underlie monogenetic disease in a patient. Subsequently, filtering of variants on the basis of a carefully considered genetic hypothesis is needed to maximize removal of false positives (removing maximum noise to work on a small manageable number of variants) while minimizing false negatives (not removing the true mutation by a too stringent filtration) and finally for properly prioritizing the remaining variants ( Figure 1).

### Genetic hypothesis

A rigorous clinical description of the IEI is paramount in the generation of a genetic hypothesis as it will influence all steps in the WES analysis: it aids grouping patients with homogenous phenotypes and hypothesizing on the potential mode of inheritance, expected allele frequency (AF), and cellular pathways that may underlie the disease.

Assuming high penetrance, analysis of a multiplex kindred (multiple patients in the same kindred) allows for the reduction of candidate variants to those shared by the diseased but not by the healthy relatives. High penetrance implies that a large proportion of individuals carrying a particular genotype also present the associated clinical trait. The same genetic homogeneity can be expected in at least a subset of unrelated patients with the same disease. As an example, 8 out of 18 families with isolated congenital asplenia (ICA) had deleterious mutations in *RPSA* whereas only one loss-of-function (LOF) mutation (p.Trp176*) has been detected in 121,156 control exomes sequenced by the Exome Aggregation Consortium (ExAC) ^14^. This enrichment of *RPSA* mutations in the ICA cohort made *RPSA* the candidate gene for ICA by genetic and phenotypic homogeneity (#1 candidate in a gene burden test) even though the mechanism of disease remains enigmatic ^15^.

Pedigree analysis also contributes to generating a hypothesis on the mode of inheritance. Consanguineous families are more likely to suffer from an autosomal-recessive (AR) inherited disease ^16^; diseases affecting only males suggest X-linked (XL) inheritance. This model can then be applied to the WES dataset: for AR disease, homozygous or compound heterozygous variants are selected; for autosomal dominant (AD) disease, heterozygous variants are selected; and for XL disease, variants called to the X-chromosome are prioritized.

### Variant-level interpretation

In IEI research, typically variants predicted to cause LOF of the studied gene are prioritized, as a defective immune response is hypothesized. LOFs are indel, frameshift, start-loss, stop-gained, missense, and essential splicing mutations. It must be kept in mind that mutations can also lead to disease if they result in a dominant negative effect (for example, interferon-gamma receptor 1 ( *IFN-γR1*) ^17^) or in a gain of function (GOF) (for example, signal transducer and activator of transcription 3 ( *STAT3*) ^18^).

Next, it is important to consider the frequency of the disease studied. Individual IEIs are rare diseases; thus, it would be highly unlikely that a disease-causing variant has an AF of more than 1% in the general population—that is, in public databases such as the Single Nucleotide Polymorphism Database (dbSNP), 1000 Genomes, ExAC, and the Genome Aggregation Database (gnomAD). The last of these also includes control WGS data ^14^.

An additional parameter to select a candidate variant is its predicted deleteriousness. Various predictions tools have been developed to calculate the effect of a nucleotide change on a gene: the sorting intolerant from tolerant (SIFT) score ^19^, the polymorphism phenotyping v2 (PolyPhen2) score ^20^, and the Combined Annotation Dependent Depletion (CADD) score ^21^, which adds information on evolutionary conservation, gene regulation, and transcription to the SIFT and PolyPhen2 calculations. Typically, disease-causing variants that are predicted deleterious by SIFT and PolyPhen or that have a CADD score above 15 (or both) are prioritized in the WES analysis. Yet false-negative rates for all three methods’ fixed genome-wide cutoffs are high ^22^. Also, deleteriousness does not imply pathogenicity: it is indeed possible that a highly deleterious variant does not explain the patient’s phenotype, for example, as there are other pathways compensating for the defect ^23^.

### Gene-level interpretation

Some genes, such as titin ( *TTN*) and mucin 16 ( *MUC16*), harbor many rare (low AF) LOF mutations that are predicted to be deleterious in the general population. They are typically not prioritized as a candidate gene for a life-threatening condition as are many IEIs ^24^, although variants in these genes per se could be promising candidates. A measure for this principle is the gene damage index (GDI) ^25^. GDI is calculated for each human gene by summing up the CADD scores of each of its 1000 Genomes Project phase 3 alternative alleles—minor allele frequency (MAF) of less than 0.5, missense/nonsense/frameshift/in-frame indels/splice variants—normalized by the corresponding expected CADD scores for alleles with the same MAF and multiplied by the AF. The lower the GDI of a particular gene, the lower the accumulated damage prediction score of the alleles in this gene are reported in the general population. The mutation significance cutoff (MSC) uses the CADD/PolyPhen2/SIFT scores of all known disease-causing mutations for each protein-coding human gene from the Human Gene Mutation Database (HGMD) and ClinVar database ^26, 27^ and generates the lowest predicted clinically/biologically relevant CADD/PolyPhen2/SIFT cutoff value for a specific gene to enable safe removal of benign variants from NGS data. A variant with a CADD score under the MSC is likely benign (even if the CADD score is greater than the popular cutoffs of 15 or 20) and can be safely removed as the true positive rate is 98% ^22^. For example, a gene with a CADD-based 99% confidence interval MSC cutoff of 25 and a variant in this gene having a CADD score of 23 can be safely filtered out. Conversely, when a gene has a CADD-based 99% confidence interval MSC cutoff of 5 and a variant in this gene has a CADD score of 7, the variant should not be filtered out on the basis of CADD score. Lastly, the information already available on the pathogenicity of a gene must be taken into account. If a gene is known to underlie an IEI and the patient’s phenotype corresponds to the phenotype previously described, the variant can be disease-causing even if the damage prediction is low ^28^. Also, as has been shown for the Toll-like receptor 3 (TLR3) pathway, mutations in genes that are biological interaction partners can lead to the same disease, in this case herpes simplex encephalitis (HSE). The Human Gene Connectome has been developed to calculate the biological distance and route from a core gene (typically a known IEI gene) to other genes: 95% of all new IEIs (discovered in 2014) are within the top 1% of at least one known IEI gene ^29^.

### Functional validation

After confirmation of the candidate disease-causing variant by Sanger sequencing, the final but crucial step is its functional validation. This should include at least three steps:

First, the impact of the patient’s alleles on transcription and protein expression of the gene of interest needs to be tested. If an allele leads to loss of expression of the protein studied, this is a strong argument in favor of the deleteriousness/pathogenicity of the variant. Expression can be predicted using publicly available databases, such as bioGPS (mRNA) ^30^ and the Human Protein Atlas (mRNA and protein expression) ^31^.

Second, the cellular phenotype of the patient should be investigated in a relevant experimental system, preferably the patient’s cells and healthy control cells. The function of the pathway of interest in patient-derived primary cells or cell lines can be compared with that of controls given that the protein is endogenously expressed in the chosen experimental system. Ideally, a positive (known responder) and negative (known LOF) control is included in the experimental set-up. If a variant is LOF, the cellular defect can be quantified and rescued by the reintroduction of the wild-type protein.

Third, the genotype and cellular phenotype of the patient should be linked to his or her clinical presentation. This is often the most challenging step as even in an appropriate *ex vivo* system, physiological mechanisms of the organism as a whole cannot be fully mimicked.

## Remaining challenges in detecting inborn errors of immunity by whole-exome sequencing

### Whole-exome sequencing – generation and bioinformatic pipeline

WES provides information on the exonic part of the genetic code only (compare with definition above). Yet, in recent years, it has become apparent that intronic regions can harbor IEI-causing mutations too. Using WGS, Starokadomskyy *et al*. showed that a deep-intronic mutation that damages *POLA1*, encoding the catalytic subunit of DNA polymerase-alpha, underlies XL reticulate pigmentary disorder (XLPDR) ^32^. Additionally, we learned that WES does not cover all parts of the exome equally well. This is due both to the PCR capture step inducing (for example, polymerase errors) and to hybridization difficulties (for instance, of sequences with high guanine-cytosine content). Also, IEIs caused by copy number variations of a gene, such as deletion or insertion spanning a sequence longer than an amplicon, will be missed, as only the second allele will be amplified. Therefore, the biggest pitfall of WES is that the gene or mutation of interest lies outside the exome covered by the WES kit.

Second, certain genes have duplicated in evolution and resulted in pseudogenes that have a sequence that parallels the “mother” active gene yet have reduced or lost function ^33^. During the annotation step, variants in a gene can incorrectly be mapped to the pseudogene, preventing the detection of mutations in the active gene. Inhibitor of nuclear factor kappa B kinase subunit gamma ( *IKBKG*) is an example of this difficulty known to the IEI field: the gene and its pseudogene copy ( *IKBKGP*) have a complex, partially overlapping genetic sequence that is not correctly mapped by WES. Therefore, functional tests and alternative sequencing approaches to capture mutations in *IKBKG* are necessary ( Figure 1).

### Genetic hypothesis

For various IEIs, individuals with a proven pathogenic genotype have been found to be asymptomatic. This phenomenon of incomplete clinical penetrance is an important pitfall and the genetic hypothesis; especially in the AD/AR/XL hypothesis, this possibility should never be neglected. It occurs when an individual has not yet encountered the key pathogen such as Epstein-Barr virus in X-linked lymphoproliferative disease. However, it can also occur in people who have been exposed. HSE, for example, is incompletely penetrant in individuals with TIR-domain-containing adapter-inducing IFN-β (TRIF) deficiency: the pathogenic *TICAM1* mutations identified in patients with HSE have been found in their “healthy” relatives, some of whom had a serologically proven history of herpes simplex virus 1 (HSV-1) infection ^34^. Likewise, the presence of diseased female carriers in an XL disease can evoke an AD model. This has been shown for chronic granulomatous disease ^35^ and Wiskott-Aldrich syndrome ^36^ and can be explained by skewed X-inactivation of the wild-type allele resulting in haploinsufficiency: a single functional copy of the IEI gene leads to disease.

Lastly, non-Mendelian and non-monogenic forms of inheritance must be considered also when studying WES data of patients with IEI. Seemingly unaffected parents can harbor a somatic mosaicism as has been described in severe congenital neutropenia ^37^. On the other hand, the possibility of revertant mosaicism explaining the milder phenotype of some patients with severe combined immune deficiency (SCID), Wiskott-Aldrich syndrome, and XL ectordermal dysplasia and immunodeficiency must be recognized ^38^. WES has already proven useful in detecting somatic mosaicism and has great potential for the future detection of revertant mosaicism ^39^. Non-monogenic etiologies of IEIs have hardly been studied by WES to date. Yet digenic inheritance has already been identified in severe congenital neutropenia ^40^, and Timberlake *et al*. have elegantly shown the potential of WES in elucidating two-locus inheritance ^41^. Non-monogenic inheritance should be included in future genetic hypotheses, especially in patients with complex PID phenotypes. As the detection of non-monogenic etiologies of IEIs is more complex, grouping patients with similar disease to look for genetic homogeneity will become even more important.

### Variant-level interpretation

The importance of exonic variants that are not predicted to be LOF, especially synonymous variants, is increasingly being recognized. Synonymous mutations, typically with a lower CADD score than LOF variants, can affect splicing or alter the timing of cotranslational folding ^42^. A recent example is the identification of a synonymous pathogenic mutation that resulted in an alternative splicing of interleukin-7R (IL-7R) in a patient with SCID ^43^. When a variant’s AF is considered, it is important to realize that the AF varies among different ethnicities ^44^ and that some ethnic groups such as Native Americans are underrepresented in public exome/genome databases. Belkadi *et al*. therefore developed a model based on principal component analysis to identify the ethnicity of a person from his or her exome data ^45^. The ethnicity-specific AF should be used in the choice for a candidate variant. Also, in several IEIs, such as Nijmegen breakage syndrome, IFNγ-R1 deficiency, and FOXN1-deficient SCID, a founder mutation has been identified ^46– 48^: the same mutation on the same allelic background is found in all patients. Typically, the frequency of carriers in the founder population is much higher than that in publically available databases.

For the interpretation of the deleteriousness prediction scores, one should realize that some genes have altered reading frames or are alternatively spliced, resulting in different isoforms. WES data are “called” to the canonical transcript and the CADD score is calculated on the basis of the predicted effect of the variant on the canonical isoform. However, it has been shown that patients with mutations in HCLS1-associated protein X-1 ( *HAX1*), underlying severe congenital neutropenia, have a different phenotype depending on the isoform that is mutated ^49^. Thus, when a genetic hypothesis is made on the basis of the phenotype, it is important to consider that the impact of a variant might be different from the effect predicted computationally on the basis of the canonical transcript.

### Gene-level interpretation

First, a constraint of the currently available deleteriousness prediction models, and also of the MSC and GDI, is that predictions are based on data on the full gene while the tolerance to functional genetic variations can vary per exon or region of a gene. Gussow *et al*. therefore introduced the sub-region Residual Variation Intolerance Score (subRVIS) ^50^. Further research into predicting sub-region and domain-specific deleteriousness is urgently needed.

Second, the MSC is dependent on published data. With the report of pathogenic mutations in a specific gene, the MSC may change and the gene is predicted to be less or more tolerant to mutations.

Third, genes are expressed differently throughout tissues. Therefore, it is possible that a protein encoded by the same gene has alternative, organ-specific interaction partners. This information was recently made available in the Genome-scale Integrated Analysis of gene Networks in Tissues (GIANT), which allows functional networks to be built around a core gene capturing its tissue-specific functional interactions ^51^. Lastly, for known IEI genes, the originally described phenotype can expand or change through WES research. After the publication of IFN-stimulated gene 15 (ISG15) deficiency as a novel genetic etiology of Mendelian susceptibility to mycobacterial disease, the phenotype was expanded to include cerebral calcifications and auto-inflammation by WES identification of additional *ISG15*-mutated individuals ^52, 53^. Thus, Mutations in known IEI genes with an (at first sight) unrelated phenotype should not per se be excluded.

### Functional validation

When human disease is studied *in vitro* or *in vivo*, be it in (patient-derived) cell lines or animal models, the limitations of the system used should be considered. Fibroblasts have proven useful to study viral susceptibility. The alleles of the first reported patients with TLR3 deficiency have been studied in depth in SV40 and primary fibroblasts ^54^. Yet, recently, it has been shown that the impaired intrinsic immunity to HSV-1 in TLR3 deficiency can be more precisely studied in TLR3-deficient central nervous system (CNS) cells derived from human-induced pluripotent stem cells (iPSCs) ^55^. Hence, the field has shifted to the use of iPSC-derived CNS cells for the study of novel genetic etiologies of HSE. Also, other organ-specific IEIs with susceptibility to infection—for example, fulminant myocarditis (myocytes ^56^) and severe influenza (lung epithelial cells ^57^)—are increasingly researched in patient-specific iPSCs.

Lastly, we progressively grasp the complexity of the human immune response itself and the non-genetically encoded factors that contribute to the clinical phenotype of the patient. As an example, Israel *et al*. demonstrated that the only patient who developed staphylococcal disease out of eight patients with Toll/interleukin-1 receptor domain-containing adapter protein (TIRAP) deficiency was the one who lacked (staphylococcal) lipoteichoic acid–specific antibody (anti-LTA Ab) and for whom the adaptive immune response thus could not rescue the innate immune defect ^58^.

## Future perspectives and conclusion

We are only beginning to appreciate the full potential of WES. In addition to the detection of the monogenic etiology of a specific IEI and modifier genes or variants in mutations that show incomplete penetrance, WES could be applied for detecting mutations in genes belonging to the same pathway in patients with the same disease. An example is the TLR3 pathway. Currently, all genes of the pathway have been identified individually. It is not unthinkable that, with the use of the human gene connectome and by combining a large set of exome data from patients with HSE, novel genes in the TLR3 pathway or patients with bigenic TLR3 pathway defects can be identified. This approach can also be envisioned for other “pathway” diseases such as Mendelian susceptibility to mycobacterial disease (IL-12/23R - IFNγ – JAK/STAT), chronic mucocutaneous candidiasis (CMC) (Th17 circuit), and the detection of novel circuits that are currently not associated with a phenotype.

A second route that is largely unexplored is the genetics of resistance against infection ^59^. Although it will be harder to prove exposure to a specific pathogen, WES can also be used to identify variants that render the host immune to a specific pathogen as has been shown for the CCR5-delta32 mutation in HIV resistance ^60^ and the sickle cell allele in malaria ^61^.

Third, the genetic understanding and detection of IEI open the way to specific therapy. A first success has been booked by the introduction of the JAK 1/2 inhibitor ruxolitinib in the treatment of CMC and autoimmunity in patients with STAT1 GOF mutations ^62^. Another mechanism of disease-specific interventions might be gene therapy such as that for patients with SCID due to adenosine deaminase–deficiency. In the far future, one can even foresee the introduction of CRISPR-Cas9 (clustered regularly interspaced short palindromic repeats–associated protein-9 nuclease) modification of blood cell DNA into clinic. IEIs would be the ideal candidates for CRISPR-Cas9 application as they are rare, monogenic defects that often can be importantly improved by a partial reconstitution, as is the case naturally in revertant mosaicism.

In conclusion, this review shows the unique potential of WES to detect known and novel IEI-causing mutations. We also demonstrate the challenges for the clinical and scientific use of the method, how these hurdles are currently being addressed, and major areas open to future research.

## Abbreviations

AD, autosomal dominant; AF, allele frequency; AR, autosomal recessive; BAM, Binary Alignment/Map; BTK, Bruton tyrosine kinase; CADD, Combined Annotation Dependent Depletion; CMC, chronic mucocutaneous candidiasis; CNS, central nervous system; CRISPR-Cas9, clustered regularly interspaced short palindromic repeats–associated protein-9 nuclease; ExAC, Exome Aggregation Consortium; GDI, gene damaging index; GOF, gain of function; HSE, herpes simplex encephalitis; HSV-1, herpes simpex virus-1; ICA, isolated congenital asplenia; IEI, inborn errors of immunity; IFN-γR1, interferon-gamma receptor 1; IKBKG, inhibitor of nuclear factor kappa B kinase subunit gamma; iPSC, induced pluripotent stem cell; ISG15, interferon-stimulated gene 15; JAK 1/2, Janus kinase 1/2; LOF, loss of function; MAF, minor allele frequency; MSC, mutation significance cutoff; NGS, next-generation sequencing; PCR, polymerase chain reaction; PID, primary immune deficiency; PolyPhen2 score, polymorphism phenotyping v2 score; SCID, severe combined immune deficiency; SIFT score, sorting intolerant from tolerant score; TLR3, Toll-like receptor 3; VCF, variant call format; WES, whole-exome sequencing; WGS, whole-genome sequencing; XL, X-linked.
